# The Effect of Ratio and Interval Training on Pavlovian-Instrumental Transfer in Mice

**DOI:** 10.1371/journal.pone.0048227

**Published:** 2012-10-29

**Authors:** Brian J. Wiltgen, Courtney Sinclair, Chadrick Lane, Frank Barrows, Martín Molina, Chloe Chabanon-Hicks

**Affiliations:** 1 Center for Neuroscience, Department of Psychology, University of California Davis, Davis, California, United States of America; 2 Department of Psychology, University of Virginia, Charlottesville, Virginia, United States of America; University of Chicago, United States of America

## Abstract

Conditional stimuli (CS) that are paired with reward can be used to motivate instrumental responses. This process is called Pavlovian-instrumental transfer (PIT). A recent study in rats suggested that habitual responses are particularly sensitive to the motivational effects of reward cues. The current experiments examined this idea using ratio and interval training in mice. Two groups of animals were trained to lever press for food pellets that were delivered on random ratio or random interval schedules. Devaluation tests revealed that interval training led to habitual responding while ratio training produced goal-directed actions. The presentation of CSs paired with reward led to positive transfer in both groups, however, the size of this effect was much larger in mice that were trained on interval schedules. This result suggests that habitual responses are more sensitive to the motivational influence of reward cues than goal-directed actions. The implications for neurobiological models of motivation and drug seeking behaviors are discussed.

## Introduction

Humans and animals learn about the consequences of their actions via instrumental conditioning [1]. This process increases the probability of adaptive behavior through reward learning and reduces inappropriate responding via punishment [2]. The ability to associate our actions with the outcomes they produce (i.e. goal-directed learning) allows us to select responses that are appropriate for particular situations and motivational states. Although many of our behaviors are initially goal-directed, they become habitual when continuously rewarded [3], [4]. Unlike actions, habits are not controlled by their consequences and tend to be automatically and reflexively elicited by stimuli in the environment.

The transition from action to habit is normally adaptive, as it allows reliably reinforced behaviors to become efficient and automatic [5]. However, this process can also be maladaptive in situations like those leading to drug addiction [6]–[9]. Drug seeking is initially goal-directed and maintained by the rewarding effects produced by drugs of abuse. However, after repeated experiences behavior becomes automatic and independent of its consequences. This fact explains many features of drug addiction that make it difficult to overcome. For example, drug-seeking behavior persists even when drugs are no longer rewarding to the addict and instead produce many unwanted, aversive consequences [8], [10].

Another feature of addiction is that abstinence is very difficult to maintain. Two motivational factors are thought to contribute to this: exposure to drug-related cues and time-dependent increases in craving and desire that accompany withdrawal (i.e. incubation) [6], [10]. These motivational processes can be modeled in animals, which has led to a detailed characterization of the anatomical circuits and the cellular and molecular mechanisms that underlie the motivation of goal-directed actions [6]–[8], [10]–[12]. However, much less is known about the effects of reward cues on habitual responding [13]. This is a critical gap in our knowledge, as addictive behavior in humans is largely characterized by automatic, stimulus controlled, habit-like responding [6], [10]. Therefore, the goal of the current study was to determine if habitual behaviors are more strongly motivated by reward cues than goal-directed actions.

Actions and habits can easily be studied in rodents. Habits are defined as instrumental behaviors that are insensitive to changes in reward value. For example, devaluing a reward by pairing it with illness will reduce instrumental actions but not habits that produce the same reward [14]–[16]. Habits often develop with time and can be fostered with specific training procedures. For example, ratio schedules, where reward delivery is contingent on the number of responses made, promote the development of action-outcome associations. In contrast, interval schedules, where reward delivery is contingent on responding after a specific amount of time has passed, promote the development of habits [17]–[19]. In the current experiments, mice were trained to lever press for food reward that was delivered after a certain number of responses had been made (i.e. ratio schedule) or after a certain amount of time had elapsed since the previous reward (i.e. interval schedule). Each of these procedures is described in detail below. After confirming that these schedules led to actions and habits, respectively, we then examined the impact of reward cues on responding.

## Materials and Methods

### Subjects

Twenty-three adult male B6129 F1 hybrid mice from Taconic were used in these studies. Throughout the experiment, mice were kept in a temperature-controlled vivarium on a 12-hour light:dark cycle and housed two animals per cage. Behavioral procedures were conducted during the light phase of the cycle. Subject weights were maintained at ∼ 85% of their original free-fed weight throughout the experiments. All experimental procedures were approved by the University of Virginia Animal Research Committee.

### Equipment

Mice were trained in eight operant conditioning chambers (Med Associates; East Fairfield, VT, USA) housed in sound and light attenuating cubicles. Each cubicle contained an exhaust fan that was on during all sessions to increase air circulation and reduce background noise. Each operant chamber contained two retractable levers mounted on the same wall, with the food magazine located in-between them. Each lever controlled a pellet dispenser that released 20 mg dustless precision pellets (Bio-Serv; Frenchtown, NJ) into the magazine. The left lever produced grain pellets while the right lever produced chocolate pellets. All chambers were connected to a central computer running MED PC software (Med Associates; East Fairfield, VT, USA), which controlled the boxes during experimental sessions and automatically recorded the data.

### Magazine Training

All mice received 2 days of magazine training for familiarization with reward delivery. Each day consisted of a 30-minute training session, during which the levers remained withdrawn and pellets were dispensed into the magazine on independent random interval schedules (60 s).

### Lever Press Training

Following magazine training, mice were split into ratio and interval groups and trained to lever press for the delivery of the two food rewards. For all animals, left lever presses led to a grain pellet reward and right lever presses led to a chocolate pellet reward. Mice underwent 2 lever-press training sessions per day, one session for each lever, spaced at least 1 hour apart. The order of these sessions was reversed each day. Each session lasted either until animals received 20 reinforcers or a maximum time of 30 minutes was reached. A continuous schedule of reinforcement, where each lever press produced a reward delivery, was used for the first 6 days of training in all animals (data not shown). Following initial lever press acquisition, the two groups of animals continued instrumental training on two different reward delivery schedules. One group was trained to lever press on a random ratio (RR) schedule of reinforcement while the other group was trained on a random interval schedule (RI) of reinforcement. The RR group underwent 2 days of ratio training on a RR5 schedule, where the probability of reinforcement for each lever press was 0.2. During the same 2 days, the RI group underwent interval training on a RI15 schedule, where the average time between reinforcer availability was 15 seconds. This was followed by 2 days of a RR10 schedule for the RR group (probability of reinforcement, P = 0.1) and a RI30 schedule for the RI group (reinforcers available every 30 s on average) and then 10 days of a RR20 schedule for the RR group (probability of reinforcement, P = 0.05) and a RI60 schedule for the RI group (reinforcers available every 60 s on average).

### Devaluation Test

A devaluation test was performed on all animals 24-hours after their last day of instrumental training. Half of the animals in each group were given ad libitum access to the grain pellet for one hour while the other half of the animals were given ad libitum access to the chocolate pellet for the same amount of time. One mouse from the ratio group did not consume pellets during this period and was excluded from analysis. Immediately following this hour of outcome devaluation, animals were placed in the operant chambers for a 2-lever choice extinction test that lasted 10-minutes. Activity on both levers was recorded.

### Pavlovian Conditioning

Following devaluation, mice received 8 days of Pavlovian conditioning. Each day consisted of a single hour-long session in which two conditional stimuli [(tone (85 dB, 2000 Hz) or white noise (80 dB)] were paired with reinforcement. The levers were retracted during these sessions. Each stimulus was presented four times during each session. The duration of each CS was 2 min, during which time reinforcement was delivered on a random interval schedule (30 s). The time between stimulus presentations was ∼5 minutes. For all animals, white noise presentations were paired with grain pellet delivery and tone presentations were paired with chocolate pellet delivery. During these sessions, head-entry detectors recorded the number of magazine entries (the conditional response) for each animal during the 2 minutes prior to stimulus presentation as well as during CS presentation. The data are expressed across training days as an elevation ratio (entries per minute during cue presentations/entries per minute during the ITI).

### Pavlovian Instrumental Transfer (PIT) Test

One day after the last Pavlovian conditioning session animals were placed in the operant chambers for a 45-minute 2-lever choice extinction test. Following an initial 8-minute baseline period, the 2 conditioned stimuli from Pavlovian training were presented intermittently to the animals. Each stimulus was presented a total of 4 times separated by ∼5-minute ITI period. Lever presses were recorded during the CS presentations and during the ITI. Rates of responding were analyzed for the lever leading to the same outcome as the CS and the lever leading to a different outcome than the CS. After this test, the animals received an additional 8 days of Pavlovian training and a second PIT test was performed, identical to the first.

## Results

**Figure 1:**
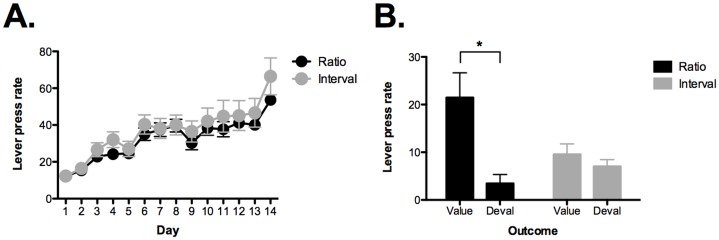
Interval training leads to habitual responding. A) Mice were trained to lever press for food pellets across 14 days. On days 1–2 the ratio group was trained on a RR5 schedule while the interval group was trained on a RI15 schedule. On days 3–4 the ratio group was trained on a RR10 schedule while the interval group was trained on a RI30 schedule. On days 5–14 the ratio group was trained on a RR20 schedule while the interval group was trained on a RI60 schedule. Both groups showed an increase in responding across training days. B) Mice trained on RR schedules showed a selective reduction in responding on the lever leading to the devalued outcome indicating that behavior was goal-directed. Mice trained on RI schedule did not exhibit a selective reduction in responding indicating that behavior was habitual. Error bars represent ± SEM. *p<0.05.

Mice were first trained to lever press on a continuous reinforcement schedule for 6 days. One lever produced grain pellets while the other led to chocolate pellets. After this period, mice were trained on random ratio (RR) or random interval (RI) reinforcement schedules for 14 days (Figure 1A). On days 1–2 the ratio group was trained on a RR5 schedule while the interval group was trained on a RI15 schedule. On days 3–4 the ratio group was trained on a RR10 schedule while the interval group was trained on a RI30 schedule. On days 5–14 the ratio group was trained on a RR20 schedule while the interval group was trained on a RI60 schedule. Both groups showed an increase in responding across training days (No effect of group *F* <1; main effect of day *F*_13, 273_ = 38.7, p<0.05; no group by day interaction *F* <1).

Previous work showed that interval training promotes habitual responding while ratio training produces goal-directed actions [17], [18], [20]. This fact can be demonstrated by using a devaluation procedure prior to a choice extinction test [14]. Following the last day of lever press training, we devalued one of the food pellets by giving mice ad libitum access to it for 60 minutes. After this period, a 10-minute extinction test was conducted where both levers were present but no reward was delivered. As shown in Figure 1B, mice trained on ratio schedules selectively reduced responding on the lever that was associated with the devalued outcome (main effect of lever, *F*_1, 10_ = 8.3, p<0.05). This indicates that animals learned the relationship between their actions and the outcomes they produce. In contrast, mice trained on interval schedules did not show a selective reduction in responding, which indicates that their behavior was habitual (no effect of lever, *F*_1, 10_ = 2.2, p>0.05).

**Figure 2:**
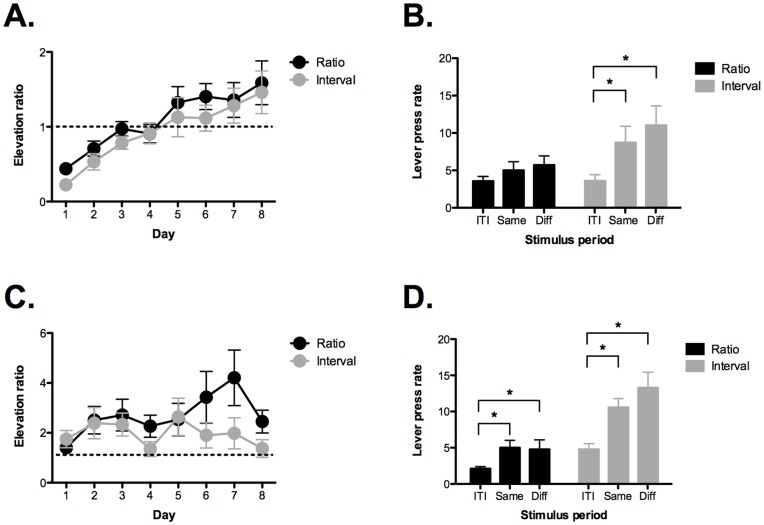
Reward cues motivate habitual responses more than goal-directed actions. A) Mice underwent Pavlovian conditioning for 8 days where conditional stimuli (CSs) were paired with the same food rewards used in instrumental training. Both ratio and interval groups showed an increase in conditional responding (magazine entries) across training days. The data are expressed as an elevation ratio  =  (CS entries/ITI entries). B) During the transfer test, mice trained on ratio schedules did not show an increase in lever pressing when the CSs were presented. In contrast, mice trained on interval schedules showed a significant increase in lever pressing during the CS presentations relative to the ITI period. This increase was not selective as it was observed on the lever leading to the same outcome as the CS and the lever leading to a different outcome (i.e. general transfer). C) Mice underwent additional Pavlovian training for 8 days. D) A second transfer test was conducted and this time mice trained on ratio schedules showed a significant increase in lever pressing when the CSs were presented. This increase was not selective and observed on both levers. Mice trained on interval schedules showed increased responding during the CS presentations that was also non-selective. The amount of transfer was significantly larger in the interval group compared to the ratio group. Error bars represent ± SEM. *p<0.05.

To examine the motivational impact of Pavlovian cues on instrumental responding, mice were trained to associate two conditional stimuli (CSs) with reward. Each CS was paired with one of the pellets used during instrumental training. Both ratio and interval groups showed an increase in conditional responding (magazine entries) across 8 training days (Figure 2A) (no effect of group, *F*_1,21_ = 1.12, p>0.05; main effect of day, *F*_7,147_ = 12.163, p<0.05; no group by day interaction, *F* <1). The data are expressed as an elevation ratio = (CS entries/ITI entries).

After Pavlovian training, the mice received a transfer test. During this test, both levers were extended but no reward was delivered. Each CS was presented 4 times and lever presses observed during the stimulus periods were compared to responding during the ITI (Figure 2B). Following 8 days of Pavlovian training, no transfer effects were observed in the ratio group. The number of lever presses was the same during the CS presentations and the ITI (no effect of stimulus period, *F*_2,22_ = 1.26, p>0.05). In contrast, the interval group showed significant transfer as lever pressing was elevated during the CS periods (main effect of stimulus period, *F*_2,20_ = 6.97, p<0.05). Post-hoc tests (Fisher’s PLSD) revealed that responding was elevated during the presentation of both CSs relative to the ITI (p values <.05). However, selective transfer was not observed as responding increased on the lever leading to the same outcome as the CS and the lever leading to a different outcome (Fisher’s PLSD, p>0.05). A direct comparison of responding during the CS periods revealed that the interval group pressed more than the ratio group when reward cues were presented (main effect of group, *F*_1,21_ = 4.12, p = 0.05). These results suggest that habits are more sensitive to the motivational impact of reward cues than goal-directed actions [13].

Using similar procedures, we previously showed that reward cues can motivate goal-directed actions in mice [21]. To determine if our current animals required more training, we conducted an additional 8 days of Pavlovian conditioning (Figure 2C). Across days, interval and ratio groups showed similar levels of magazine entries in response to the CSs (no effect of group, *F*_1,21_ = 1.07, p>0.05; main effect of day, *F*_7,147_ = 3.14, p<0.05). There was a significant group x day interaction (*F*_7,147_ = 2.3, p<0.05) but post-hoc tests (Fisher’s PLSD) found no differences between ratio and interval groups on any of the training days (all p values >.05).

After additional Pavlovian training, we observed positive transfer in both groups (Figure 2D). Mice trained on ratio schedules showed a significant increase in responding during the CS presentations (effect of stimulus period, *F*_2,22_ = 4.6, p<0.05), Post-hoc tests (Fisher’s PLSD) revealed that responding was elevated during the presentation of both CSs relative to the ITI (p values <.05). However, selective transfer was not observed as responding increased on the lever leading to the same outcome as the CS and the lever leading to a different outcome (Fisher’s PLSD, p>0.05). The interval group, once again, increased responding during both CS periods relative to the ITI (effect of stimulus period, *F*_2,20_ = 13.8, p<0.05) (Fisher’s PLSD p values <0.05). Similar to the ratio group, selective transfer was not observed as responding was equivalent during the presentation of both CSs (Fisher’s PLSD, p>0.05). Finally, a direct comparison of responding during the CS periods revealed that the interval group pressed more than the ratio group when reward cues were presented (main effect of group, *F*_1,21_ = 15.17, p<0.05). Therefore, even though positive transfer was observed in both groups the effect was larger for habits than goal-directed actions.

## Discussion

Consistent with previous studies, we found that interval training led to habitual responding while ratio training produced goal-directed behaviors [17]–[19]. This fact was demonstrated by devaluing one of the food rewards prior to a choice extinction test. In the ratio group, this procedure led to a selective reduction in responding on the lever associated with the devalued outcome. In contrast, mice trained on interval schedules responded similarly on both levers indicating that their behavior was not mediated by the knowledge of specific response-outcome associations [4].

Whether a response is encoded as an action or a habit is determined, in part, by the correlation between the behavior and the outcome it produces [3], [4]. In a ratio schedule there is a strong correlation between responding and reward. The more the animal responds the more food it gets. The less it responds, the less food it gets. On an interval schedule, responding is also required to produce food. However, there is a weak correlation between the amount of responding and the amount of reward. An animal that responds at a very high rate gets the same amount of food as an animal that responds at a low rate. As a result, the experienced correlation between response and reward on an interval schedule is very different from that experienced on a ratio schedule. Previous work has shown that lower correlations promote habit formation [4].

To determine the motivational impact of reward cues on instrumental responding we conducted a Pavlovian-instrumental transfer test in these groups. We found that reward cues exerted a stronger influence on habits than goal-directed actions. This finding is consistent with a previous study that used over-training to produce habitual responding [13]. Together, these results suggest that habits are particularly susceptible to sensory cues that are associated with reward.

Habits may have been more responsive to reward cues because our procedures produced a general form of Pavlovian-instrumental transfer. The presentation of conditional stimuli increased responding on the lever associated with the same reward and on the lever associated with a different reward. This result likely occurred because of the similarity between the rewards used in our experiments (two food pellets). We previously observed selective transfer in mice when food pellets and a sucrose solution were used [21].

General transfer is mediated by an emotional response that is common to both outcomes while selective transfer occurs when the CS activates specific sensory features of the reward [13], [22]. Habits should be particularly sensitive to general transfer effects as they are not associated with detailed sensory representations of the outcome [23]. Anatomically, the basolateral nucleus of the amygdala (BLA) is thought to encode emotional events with reference to their sensory-specific features while the central nucleus (CeA) encodes general motivational or affective significance [24]. Consistent with this framework, the BLA is required for specific transfer while general transfer involves the CeA [25], [26].

These data imply that during general transfer, activation of the CeA preferentially engages brain structures that encode habits. A recent study suggests that this may be the case. Lingawi & Balleine (2012) examined interactions between the CeA and the dorsolateral striatum (DLS), a structure that is essential for habit learning in humans and animals [3], [27], [28]. They found that disconnecting the anterior CeA and DLS prevented the acquisition of habitual responding when rats were over-trained [29]. This suggests that the CeA provides an important reinforcement signal to the DLS as habits are being acquired. In the same study, lesions of the CeA also prevented Pavlovian cues from motivating instrumental responding during a transfer test. However, the effects of disconnecting the CeA and DLS on transfer were not examined. Additional studies are therefore needed to determine if signaling between the CeA and DLS is required for reward cues to motivate habitual responding.

The current results may also be relevant to addiction, which is mediated by drug seeking responses that are automatic and insensitive to consequences [8], [10], [30]. A major source of relapse in addicted individuals, results from exposure to environments and stimuli that are associated with drugs [6], [10]. Our data suggest that the emotional impact of reward cues may be particularly strong motivators of drug seeking behavior precisely because these responses are habitual. If this is the case, then interventions that alter affective responses elicited by drug-associated stimuli should serve as particularly effective treatments.
